# Efficacy of silicone foam dressings in preventing pressure injuries in the sacral and heel areas of patients: a meta-analysis

**DOI:** 10.3389/fmed.2025.1644290

**Published:** 2025-08-13

**Authors:** Tingkui Fu, Xiuqin Wu, Biao Yu

**Affiliations:** ^1^Department of Intensive Care Unit, The Second Hospital of Nanjing, Affiliated to Nanjing University of Chinese Medicine, Nanjing, China; ^2^Department of Operating Room, The Affiliated Jiangning Hospital of Nanjing Medical University, Nanjing, China

**Keywords:** silicone foam dressings, pressure injuries, skin, care, nursing, clinical

## Abstract

**Background:**

Pressure injury prevention is clinically critical for optimizing patient outcomes. This meta-analysis systematically evaluates the efficacy of silicone foam dressings in preventing pressure injuries specifically in the sacral and heel regions.

**Methods:**

A comprehensive literature search was conducted across PubMed, Embase, ClinicalTrials.gov, the Cochrane Library, Web of Science, China National Knowledge Infrastructure (CNKI), Wanfang, and Weipu Databases, spanning from database inception to May 10, 2025. Eligible studies were randomized controlled trials (RCTs) investigating silicone foam dressings for pressure injury prevention. Meta-analyses were performed using RevMan 5.4 software.

**Results:**

Ten RCTs involving 4,817 patients were included, with 2,670 patients in the silicone foam dressing intervention group. Meta-analysis results showed that silicone foam dressings significantly reduced the incidence of stage I pressure injuries in the sacral region [relative risk (RR) = 0.18, 95% confidence interval (CI): 0.09–0.33, *p* < 0.001] and heel region (RR = 0.30, 95% CI: 0.14–0.66, *p* = 0.003). Additionally, these dressings decreased the incidence of stage II and more severe pressure injuries in the sacral region (RR = 0.42, 95% CI: 0.31–0.58, *p* < 0.001) and heel region (RR = 0.52, 95% CI: 0.27–0.99, *p* = 0.05).

**Conclusion:**

Silicone foam dressings exhibit significant efficacy in reducing pressure injury incidence in the sacral and heel regions, supporting their clinical recommendation. However, further research is required to explore their cost-effectiveness and long-term outcomes to strengthen evidence for broader implementation.

## Introduction

Pressure injury is a localized injury to the skin and underlying tissue resulting from exposure to mechanical forces, including pressure, shear, and/or friction (1). The incidence of pressure injury varies significantly between general inpatients and intensive care patients, with rates of approximately 8.66 and 30.18%, respectively (2). As a common iatrogenic complication in clinical settings, the occurrence of pressure injury not only reflects the level of nursing quality within healthcare institutions but also leads to a series of adverse clinical outcomes (3). These include prolonged hospital stays, increased medical costs, diminished patient quality of life, and in severe cases, life-threatening conditions (4, 5). Moreover, complications related to pressure injury may exacerbate patient–provider conflicts and disrupt the harmonious delivery of medical services (6). The sacral and heel regions as the highest-risk areas for pressure injury development in bedridden patients. High-risk populations include, but are not limited to, critically ill patients, those with spinal cord injuries, individuals receiving palliative care, postoperative patients, and emergency patients requiring prolonged transport (7, 8). This epidemiological profile underscores the importance of early risk assessment and targeted preventive interventions.

In clinical practice for pressure injury prevention, a range of functional dressings have been deployed, with silicone foam dressings emerging as a focus of interest owing to their distinctive structural attributes (9). Their three-dimensional architecture is engineered to conform closely to the anatomical contours of the sacrum and heels, a design feature that enhances interface compatibility with these high-risk pressure points (10). The composite absorbent core, comprising polyurethane foam, nonwoven fabric, and highly absorbent polyacrylate fibers, is integrated with a polyurethane film layer and secured via a porous silicone adhesive—elements that collectively contribute to their functional performance (11, 12). Despite these structural advantages, there remains a critical gap in the literature: systematic, evidence-based evaluations that synthesize both the clinical preventive efficacy and cost-effectiveness of these dressings are notably lacking to date.

Against this backdrop, the present meta-analysis was designed to scientifically assess the clinical utility of silicone foam dressings in preventing pressure injuries in the sacral and heel regions. By synthesizing current evidence, this study aims to provide robust, evidence-based support for clinical decision-making in dressing selection and nursing practice, thereby bridging the gap between research and practical application.

## Methods

The present study adopted a meta-analytic approach and strictly adhered to the Preferred Reporting Items for Systematic Reviews and Meta-Analyses (PRISMA) guidelines (13). Given that this study was a meta-analysis, it did not require ethical approval or patient consent.

We conducted a comprehensive literature search across multiple databases, including PubMed, Embase, ClinicalTrials.gov, the Cochrane Library, Web of Science, China National Knowledge Infrastructure (CNKI), Wanfang and Weipu Databases. The literature search was conducted across all included databases from their respective inception dates up to and including May 10, 2025. This time frame was deliberately defined to encompass the full breadth of relevant publications while establishing a clear temporal boundary: all studies meeting the inclusion criteria and published on or before this cutoff date were systematically incorporated into the analysis. For this meta-analysis, the search strategy was structured as follows: (“Pressure injury” OR “Pressure Ulcers” OR “Ulcer” OR “Bedsore” OR “Pressure Sore” OR “Pressure skin injury”) AND (“foam dressing” OR “dressing” OR “silicone foam”). To ensure methodological consistency, the core components of this Boolean strategy—encompassing key search terms, logical operators (AND/OR), and any truncation or wildcard techniques employed—were systematically standardized across all databases included in the study. This uniformity was deliberately maintained to preserve the comparability of literature retrieval across platforms, thereby minimizing potential variability in the scope or relevance of retrieved records.

The inclusion and exclusion criteria for literature in this study were defined in accordance with the PICOS framework. For the population, studies were included if they enrolled patients aged 18 years or older without pre-existing pressure injuries, with baseline characteristics comparable between the intervention and control groups. Regarding the intervention, the experimental group received silicone foam dressings, while the control group received standard care or alternative types of dressings. The primary outcomes of interest included the incidence of pressure injuries in the sacral and heel regions, as well as cost-effectiveness analyses. In terms of study design, only RCTs were included, and the language of the included literature was restricted to Chinese or English. The exclusion criteria encompassed conference abstracts, review articles, systematic reviews, or animal studies; literature for which full texts were unavailable; and duplicate publications.

During the literature screening process, two researchers independently reviewed and selected articles based on the established inclusion and exclusion criteria. Any disagreements that arose during the screening were resolved through discussion with a third researcher to reach a consensus and make the final decision. After using EndNote software to remove duplicates from the retrieved articles, the researchers first conducted an initial screening by reading the titles and abstracts, eliminating those that clearly did not meet the inclusion criteria. Subsequently, the articles that passed the initial screening were subjected to a more detailed secondary screening through full-text reading. When necessary, the researchers proactively contacted the authors of the articles to obtain crucial data information essential for the study, thereby ensuring the quality and integrity of the included articles. For the articles finally included in the study, we meticulously documented their relevant information, including the first author, year of publication, country where the study was conducted, sample size, intervention measures, assessment time points, and outcome measures.

The methodological quality of all included studies was rigorously evaluated using the Cochrane Library’s quality assessment criteria (14). These criteria comprise seven core domains: adequacy of random sequence generation, effectiveness of allocation concealment, implementation of blinding for participants and intervention providers, application of blinding for outcome assessors, completeness of outcome data, presence of selective reporting, and identification of other potential sources of bias. For each domain, a judgment of “low risk of bias, ““high risk of bias,” or “unclear risk of bias” was assigned based on the degree of adherence to methodological standards.

The assessment was performed by a team of three independent researchers. The process was designed to minimize subjectivity through a two-stage review: first, each researcher independently evaluated the included studies against the Risk of Bias criteria and assigned risk judgments (low, high, or unclear) for each domain; second, all three researchers convened to compare their assessments, resolve discrepancies through structured discussion, and reach a consensus on final judgments. This multi-author, consensus-driven approach enhances the reliability of the assessments by mitigating individual biases and ensuring consistency across evaluations. These details have been clarified in the revised methodology section.

For the meta-analysis in this study, RevMan 5.4 software was employed following standardized analytical procedures. Heterogeneity among included studies was initially assessed using the *p*-value and *I*^2^ statistics, with a fixed-effects model applied when *p* > 0.1 and *I*^2^ < 50%, while a random-effects model was utilized when *p* ≤ 0.1 or *I*^2^ ≥ 50%, accompanied by subgroup analyses to investigate potential sources of heterogeneity. Publication bias was evaluated through funnel plot visual inspection, and sensitivity analysis was conducted using the leave-one-out method to examine result robustness. The relative risk (RR) with 95% confidence intervals (CI) served as the primary effect measure, with statistical significance defined as *p* < 0.05.

## Results

**Figure 1:**
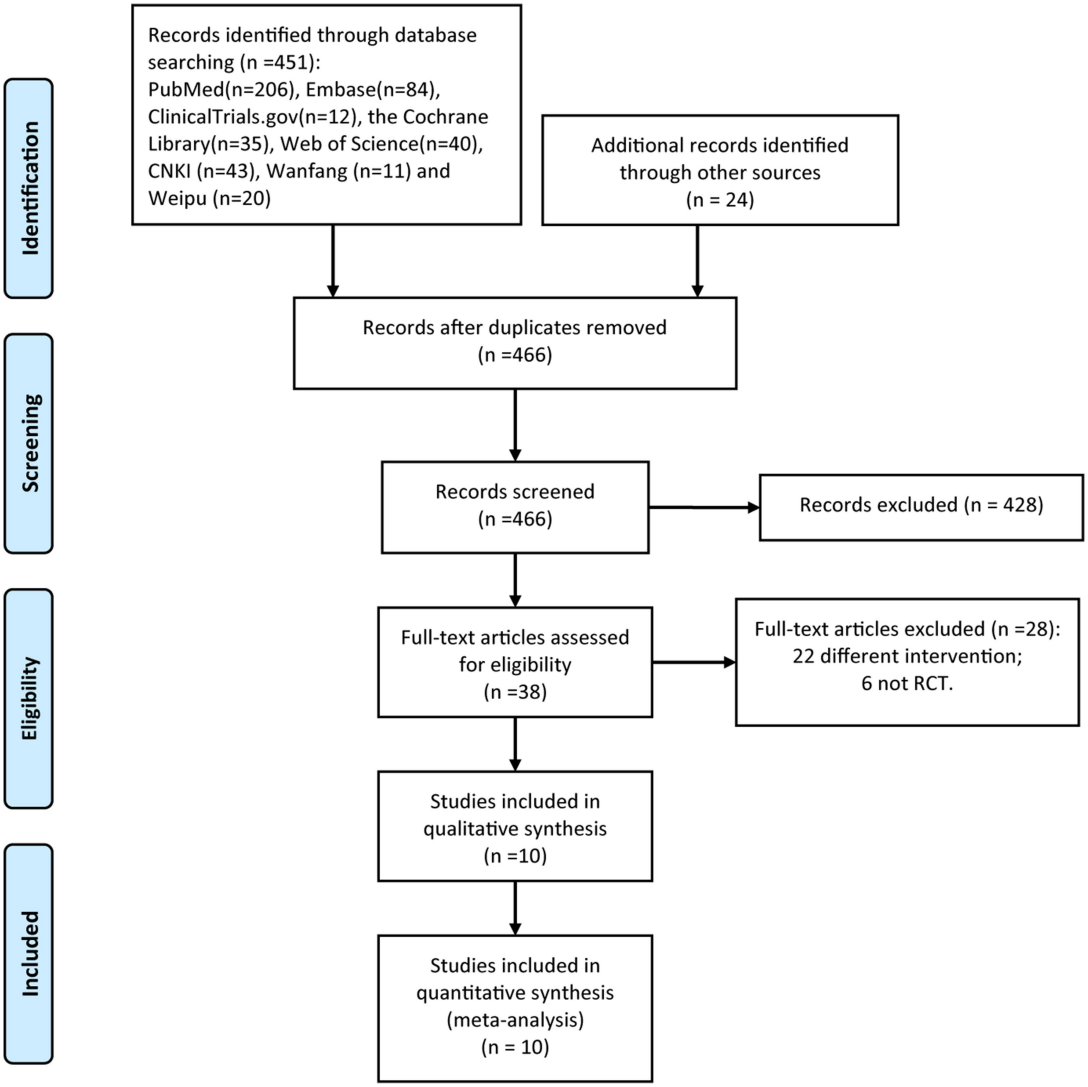
PRISMA flow diagram of RCT inclusion.

As shown in in Figure 1, the initial literature search yielded 475 potentially relevant articles. After removing duplicates using EndNote software, 466 records remained for screening. Following a rigorous review of titles and abstracts, 38 articles were preliminarily selected based on our predefined inclusion criteria. Subsequent full-text evaluation resulted in the final inclusion of 10 RCTs (15–24) for analysis.

The 10 included RCTs (15–24) comprised a total of 4,817 patients. Among them, 2,670 patients received silicone foam dressings intervention, while 2,147 patients received conventional care. The studies were reported from Australia, the United States, Germany, China, Italy, and Japan. The included studies enrolled patients with diverse primary diagnoses and characteristics, predominantly consisting of individuals with neurological conditions and postoperative patients. The populations primarily consisted of individuals with neurological conditions (e.g., spinal cord injuries, advanced neurodegenerative disorders) or postoperative patients in the acute recovery phase, where movement was restricted due to medical reasons or physical limitations. These patients typically lacked the ability to independently reposition themselves or shift their body weight—key movements that help mitigate pressure injury risk. However, it is important to note that detailed granular data on specific mobility capacities (e.g., partial movement of extremities) were not consistently reported across studies. The basic characteristics of the included studies are presented in Table 1.

**Table 1:** The characteristics of included RCTs.

RCT	Country	Sample size		Intervention		Assessment timepoint	Outcomes
		Silicone foam dressing group	Control group	Silicone foam dressing group	Control group		
Beeckman et al. (15)	Australia	1,066	539	Upon admission to the emergency department, multilayer soft silicone foam dressings were applied to each heel. After admission to the ICU, the dressings were retained with tubular bandages	Routine care	During the length of ICU stay	Incidence of sacrococcygeal and heel pressure injuries
Chao et al. (16)	China	16	16	Silicone foam dressings were applied to five areas of the patient, including both heels, the sacrococcygeal region, and the greater trochanters of both femurs	Routine care	Before discharge	Incidence of pressure injuries and skin conditions around the bony carina
Forni et al. (18)	Italy	351	358	Multilayer silicone polyurethane foam was applied to the sacrum	Routine care	Within 7 days after admission	Incidence of sacrococcygeal pressure injury
El Genedy et al. (17)	Germany	212	210	Multilayer silicone foam dressings were properly applied to the sacral and heel areas, and no other skincare products were used between the skin and the dressing. The dressings were changed regularly every 3 days	Routine care	Before discharge	Incidence of sacrococcygeal and heel pressure injuries and cost-effectiveness
Hahnel et al. (19)	Germany	212	210	Dressings were applied to the two heels and the sacral area. The dressings were changed every 3 days, and the skin beneath the dressings was checked daily	Routine care	During the length of ICU stay	Incidence of sacrococcygeal and heel pressure injuries
Kalowes et al. (20)	USA	184	182	A sacral foam dressing was applied to the sacrum and maintained throughout the patient’s ICU stay. Routine skin assessments were performed daily. The dressing was changed every 3 days or whenever it became soiled or displaced	Routine care	Before discharge	Incidence of sacrococcygeal pressure injury
Oe et al. (21)	Japan	300	300	Multilayer silicone foam dressings were applied to the sacrum and coccyx. The dressings were partially removed to assess the development of the skin and pressure ulcers. Dressings that became soiled or displaced were replaced in a timely manner	Routine care	Within 14 days	Incidence of sacrococcygeal pressure injury
Santamaria et al. (23)	Australia	161	152	Soft silicone foam dressings were applied to the sacrum and heels upon emergency admission. After admission to the ICU, the dressings were changed every 3 days	Routine care	During the length of ICU stay	Incidence of sacrococcygeal and calcaneal pressure injuries; cost-effectiveness
Santamaria et al. (22)	Australia	138	150	Multiple layers of silicone foam dressing were applied to the sacrum and each heel. The interval between dressing changes was 3 days	Routine care	After 4 weeks of follow-up	Incidence of sacrococcygeal and heel pressure injuries
Wang et al. (24)	China	30	30	During surgery, foam dressing was placed on the sacrococcygeal region	Routine care	2 h after surgery	Skin redness area and sacrococcygeal redness resolution time

**Figure 2:**
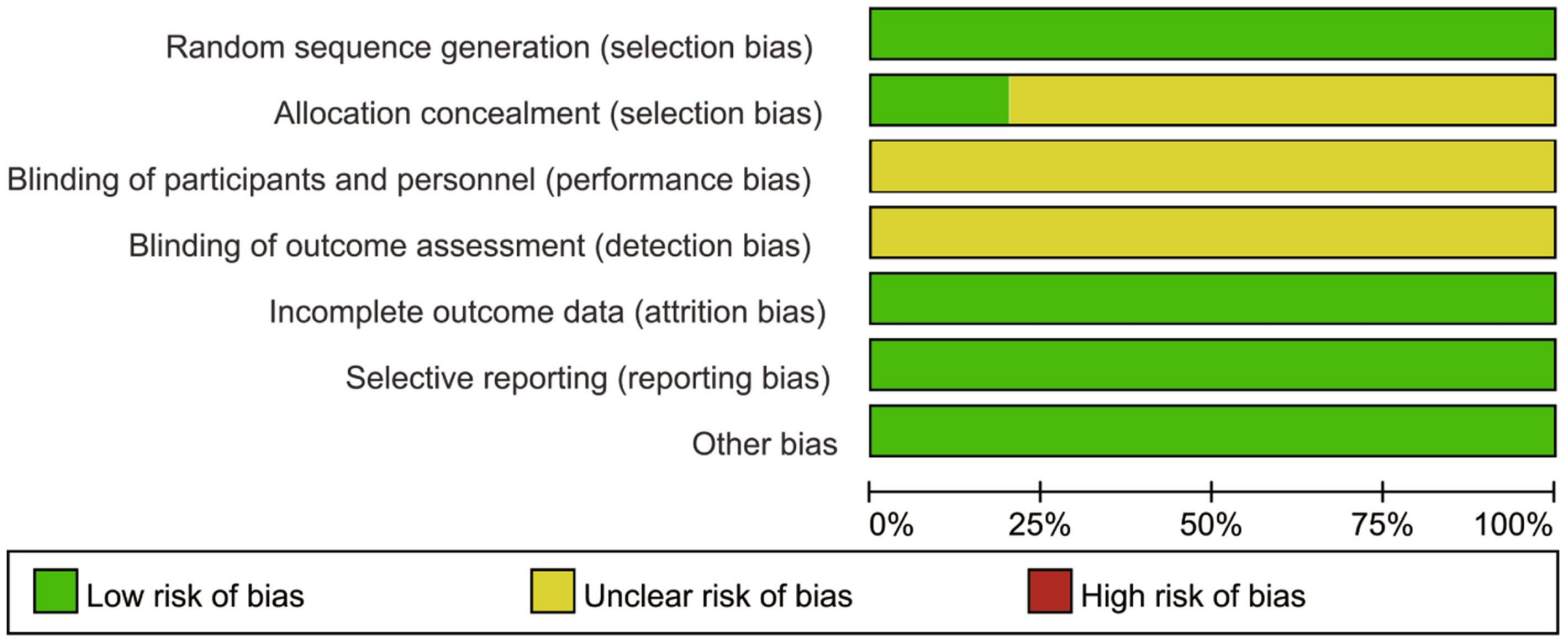
Risk of bias graph.

**Figure 3:**
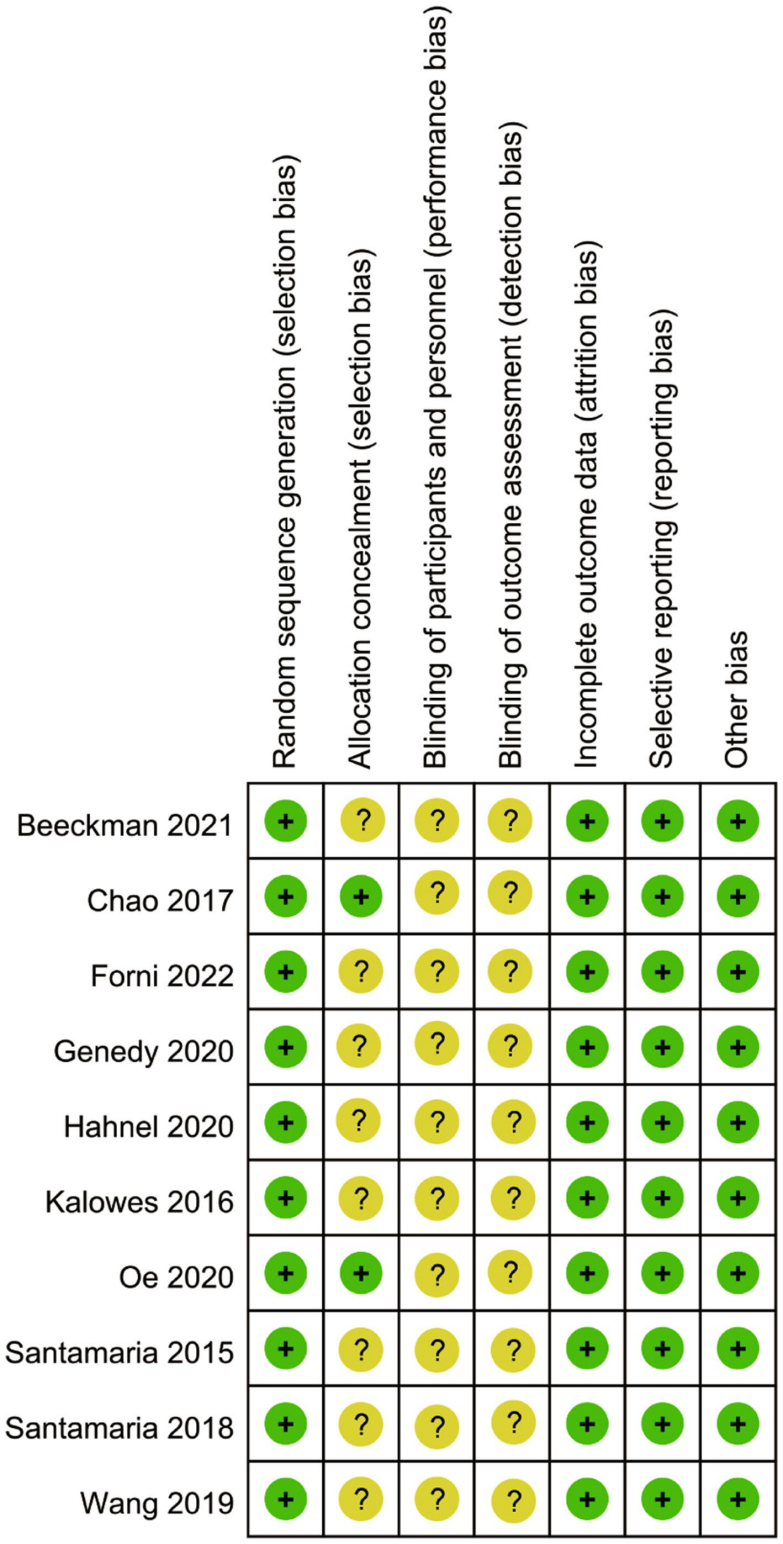
Risk of bias summary.

As depicted in Figures 2, 3, the overall quality of the 10 included RCTs is deemed to be satisfactory. In terms of randomization, all 10 RCTs provided detailed descriptions of the methods used to generate random sequences, thereby ensuring the random allocation of study participants. However, with regard to allocation concealment, only two studies explicitly outlined the specific methods employed. The remaining studies failed to mention any details regarding the implementation of allocation concealment, which may potentially compromise the objectivity of the study results. Moreover, none of the studies reported on the blinding arrangements for participants, intervention providers, or outcome assessors, which could increase the risk of the study results being influenced by subjective factors. Despite these limitations, all included studies meticulously reported data on the primary outcome measures, and no other potential sources of bias were identified during the data collection and reporting processes.

**Figure 4:**
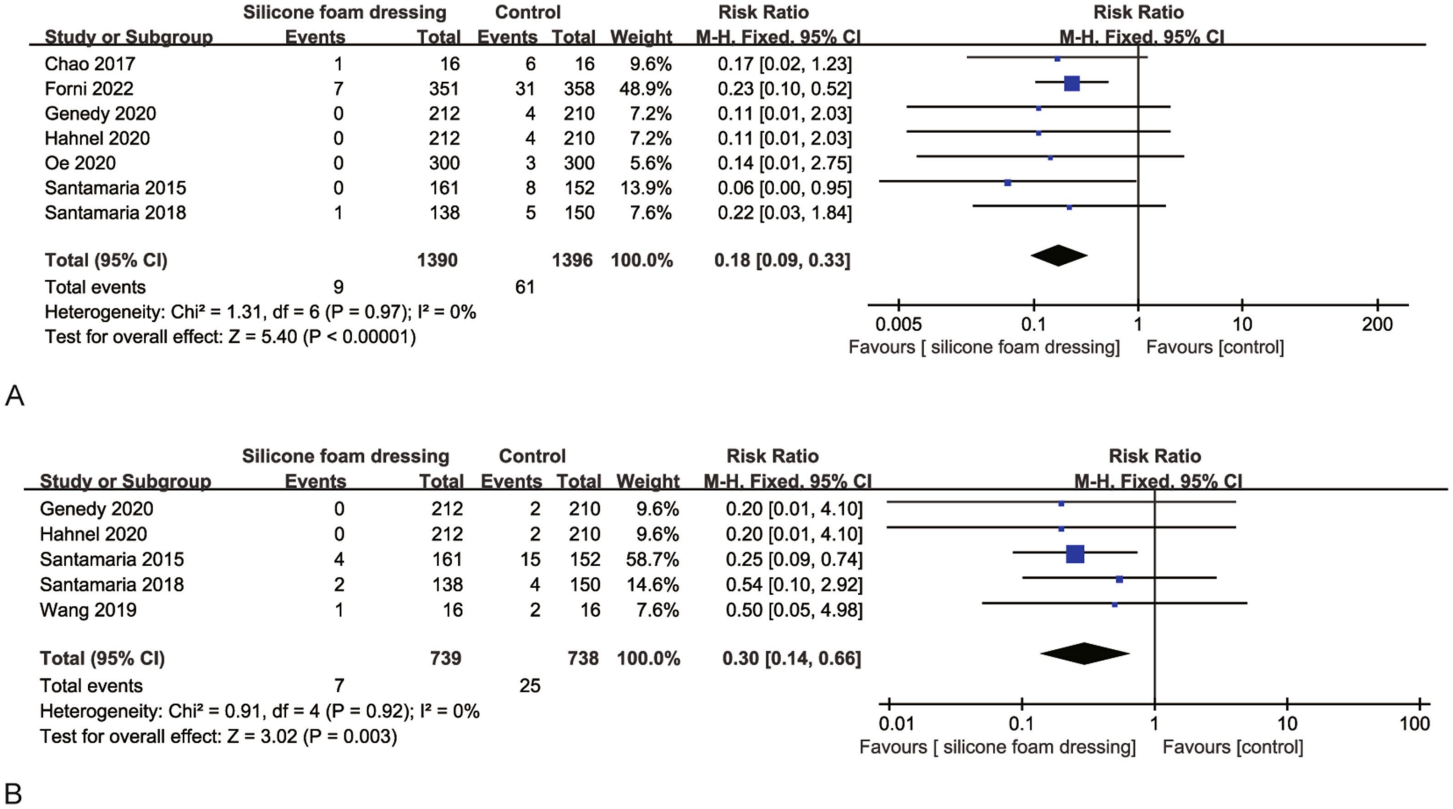
Forest plots of the efficacy of silicone foam dressings in preventing stage I pressure injuries in the sacral or heel area of patients. **(A)** Forest plot of the efficacy of silicone foam dressings in preventing stage I pressure injuries in the sacral area of patients. **(B)** Forest plot of the efficacy of silicone foam dressings in preventing stage I pressure injuries in the heel area of patients.

A pooled analysis of seven RCTs consistently evaluated the efficacy of silicone foam dressings in preventing stage I pressure injuries at the sacral region. Heterogeneity assessment revealed excellent inter-study consistency (*p* = 0.97, *I*^2^ = 0), warranting the use of a fixed-effects model for meta-analysis. The synthesized results demonstrated a statistically significant risk reduction for stage I sacral pressure injuries with silicone foam dressings compared to standard care (RR = 0.18, 95% CI: 0.09–0.33, *p* < 0.001), as illustrated in Figure 4A. These findings suggest that the clinical application of silicone foam dressings may provide effective prophylaxis against the development of stage I pressure injuries in the sacral area.

The meta-analysis incorporated five RCTs that evaluated the effect of silicone foam dressings on the incidence of stage I pressure injuries in the heel region. Heterogeneity testing demonstrated high homogeneity among the included studies (*p* = 0.92, *I*^2^ = 0), supporting the use of a fixed-effects model for analysis. The pooled results revealed that silicone foam dressings significantly reduced the risk of developing stage I heel pressure injuries compared to control interventions (RR = 0.30, 95% CI: 0.14–0.66, *p* = 0.003), with this difference reaching statistical significance (see Figure 4B). These findings suggest that the clinical application of silicone foam dressings may be an effective preventive measure against stage I pressure injuries in the heel area.

**Figure 5:**
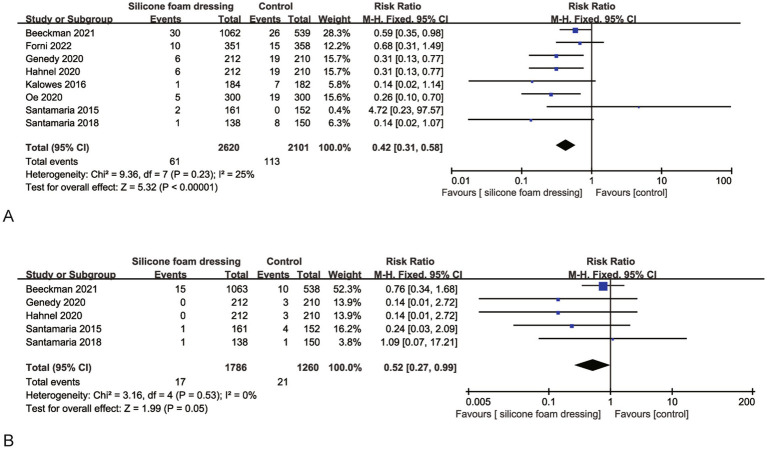
Forest plots of the efficacy of silicone foam dressings in preventing stage II and more severe pressure injuries in the sacral or heel area of patients. **(A)** Forest plot of the efficacy of silicone foam dressings in preventing stage II and more severe pressure injuries in the sacral area of patients. **(B)** Forest plot of the efficacy of silicone foam dressings in preventing stage II and more severe pressure injuries in the heel area of patients.

Eight RCTs evaluated the impact of silicone foam dressings on the incidence of stage II and more severe pressure injuries in the sacral region. Heterogeneity assessment revealed substantial homogeneity among studies (*p* = 0.53, *I*^2^ = 25%), justifying the application of a fixed-effects model for meta-analysis. The pooled results demonstrated that silicone foam dressings significantly reduced the risk of developing stage II and more severe pressure injuries compared to standard care (RR = 0.42, 95% CI: 0.31–0.58, *p* < 0.001), with this protective effect reaching statistical significance (Figure 5A). These findings indicate that silicone foam dressings may serve as an effective preventive intervention against stage II and more severe pressure injuries in clinical practice.

Five RCTs were systematically analyzed to evaluate the efficacy of silicone foam dressings in preventing stage II and more severe pressure injuries in the heel region. The heterogeneity test demonstrated excellent consistency across studies (*p* = 0.53, *I*^2^ = 0%), supporting the use of a fixed-effects model for meta-analysis. The pooled results indicated a statistically significant reduction in the risk of developing stage II + pressure injuries with silicone foam dressings compared to control groups (RR = 0.52, 95% CI: 0.27–0.99, *p* = 0.05), as detailed in Figure 5B. These findings suggest that the clinical implementation of silicone foam dressings may provide effective prevention against advanced-stage pressure injuries in the heel area.

**Figure 6:**
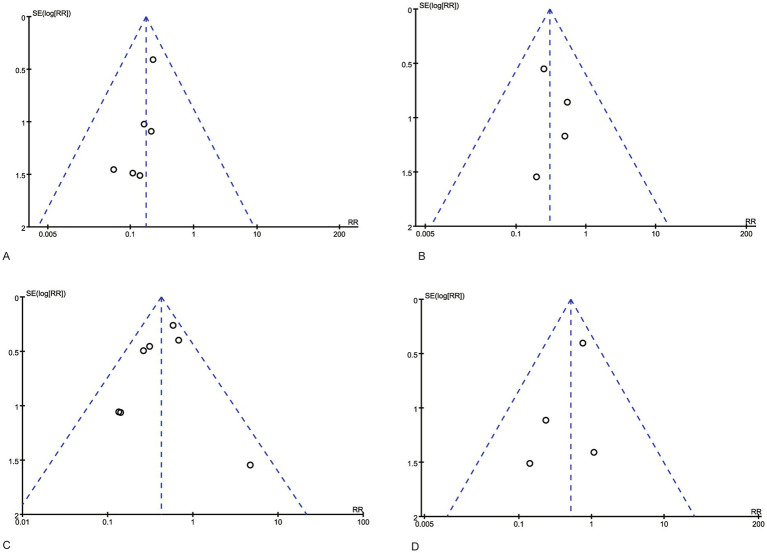
Funnel plots of the efficacy of silicone foam dressings. **(A)** Funnel plot of the efficacy of silicone foam dressings in preventing stage I pressure injuries in the sacral area of patients. **(B)** Funnel plot of the efficacy of silicone foam dressings in preventing stage I pressure injuries in the heel area of patients. **(C)** Funnel plot of the efficacy of silicone foam dressings in preventing stage II and more severe pressure injuries in the sacral area of patients. **(D)** Funnel plot of the efficacy of silicone foam dressings in preventing stage II and more severe pressure injuries in the heel area of patients.

The funnel plot analysis (Figure 6) revealed that the included studies exhibited symmetry, with all studies positioned below the inverted funnel. This suggests a low likelihood of publication bias. To verify the robustness of the study results, we employed two methods for sensitivity analysis. First, the effect size was combined using a fixed-effect model and a random-effects model to verify the results. Second, the influence of each study on the overall effect size was systematically assessed by excluding each study one by one. The analysis results showed that the combined effect size did not change significantly under different models and after excluding any single study, which confirmed the high stability of the meta-analysis results.

Egger’s test results demonstrated no evidence of publication bias across the synthesized outcomes of the included studies, with all *p*-values exceeding 0.05.

## Discussion

The results of this meta-analysis demonstrate that silicone foam dressings can significantly reduce the overall incidence of pressure injury of all stages in the sacral and heel regions. Existing research evidence indicates a significant correlation between the occurrence of stage I pressure injury and stage II pressure injury (25). It has been found that stage I pressure injury can increase the risk of subsequent stage II pressure injury by nearly threefold (26). From a pathophysiological perspective, the non-blanchable erythema characteristic of stage I pressure injury often signifies local tissue microcirculatory impairment and a higher risk of progression to more severe injuries. Therefore, early identification and intervention of stage I pressure injury hold significant clinical value in preventing the progression of injuries (27). Although multiple high-quality studies have confirmed that silicone foam dressings can effectively reduce the incidence of stage I pressure injury in the sacral and heel regions, it is important to emphasize that such preventive dressings should be used as a supplement to, rather than a replacement for, standard preventive measures. According to international guidelines, basic preventive measures include, but are not limited to, regular repositioning, nutritional support, skin assessment, and the use of pressure-relieving devices, which remain the core strategies for pressure injury prevention (28, 29). By explicitly advocating for long-term cost-effectiveness investigations that span patient, institutional, and community levels, this study contributes a nuanced perspective: it not only validates the clinical utility of silicone foam dressings but also articulates a roadmap to address the evidence gap in holistic value assessment. Such advancements are pivotal for translating efficacy data into actionable insights, enabling more informed decision-making in resource allocation and the development of cost-sensitive, patient-centered care strategies. Our study differentiates itself by its targeted focus on high-risk anatomical sites, its critical synthesis of existing cost-effectiveness evidence, and its proactive delineation of future research priorities—collectively strengthening the bridge between clinical efficacy and real-world implementation.

Our finding indicates that silicone foam dressings are highly effective in reducing the incidence of stage II and higher pressure injury in the sacral region. Their mechanism of action is primarily manifested in three aspects: Firstly, in acute care units and intensive care units (ICU), the dressings redistribute pressure by increasing the area over which force is applied. This significantly reduces local tissue pressure (by up to 30–40%) and shear forces, thereby effectively preventing the occurrence of hospital-acquired pressure injury (30). Secondly, considering the anatomical location of the sacral region (adjacent to the perianal area), the waterproof and breathable properties of the dressings (with a breathability rate of >1,000 g/m^2^/24 h) form an effective physical barrier (31). This prevents skin maceration and infection caused by fecal contamination. Research data show that the use of silicone foam dressings can reduce the incidence of incontinence-associated dermatitis by over 60% (32). It is important to note that the application of dressings must be accompanied by a standardized skin cleaning protocol (using pH-balanced cleansers) and timely dressing changes (immediate replacement when contaminated or displaced) (33).

The heel region represents a particularly vulnerable anatomical site for pressure injury development due to its distinct pathophysiological characteristics. Anatomically, the heel’s pronounced curvature and prominent bony contours create uneven pressure distribution, with particularly high pressure gradients over the calcaneal prominence. Crucially, the heel’s subcutaneous tissue consists primarily of a fat pad with minimal muscular cushioning, rendering it highly susceptible to tissue distortion and shear deformation under mechanical loading. The heel tissue may undergo 30–40% deformation under body weight—substantially higher than other anatomical sites (e.g., 20–25% in the sacral region) (34). This excessive mechanical stress induces microcirculatory impairment, leading to localized ischemia and cellular damage (35). Our meta-analysis provides robust evidence supporting the clinical efficacy of silicone foam dressings in heel pressure injury prevention. The protective efficacy of silicone foam dressings is achieved through three interrelated biomechanical and microclimate regulatory mechanisms: optimal material compliance ensures anatomical conformity and effective pressure redistribution by adapting to the heel’s contours, while the architecture provides substantial shear force absorption (approximately 70%) through its viscoelastic properties (36); concurrently, the dressing’s superior moisture vapor transmission rate (>1,000 g/m^2^/24 h) maintains an optimal microclimate by balancing humidity and temperature, thereby addressing both mechanical and environmental risk factors for pressure injury development in a synergistic manner (37). For maximal preventive efficacy, dressing application should be integrated into a comprehensive prevention protocol incorporating 30° lateral positioning and heel pressure-relief devices (38). This multimodal approach has received Grade A recommendation in the recent international guidelines (39).

The absence of reported data on anti-decubitus air mattress use in the included studies represents a notable gap, as these devices—through dynamic pressure redistribution—are recognized as a cornerstone of pressure injury prevention in acute and intensive care settings, potentially confounding the observed effects of silicone foam dressings. While theoretical and empirical evidence suggests that anti-decubitus air mattresses may act as adjuvant interventions by reducing tissue interface pressure, their synergistic or additive effects with silicone foam dressings remain unquantified in the current dataset, limiting conclusions about combined prophylactic efficacy. This lack of reporting underscores the need for future studies to explicitly document concurrent use of pressure-redistribution devices, as such data would enable more precise attribution of pressure injury prevention outcomes to specific interventions and clarify the role of multi-modal strategies in high-risk populations.

The economic burden of pressure injury on the healthcare system is substantial and cannot be overlooked. Cost-effectiveness assessments must account for the variability in clinical settings. Existing studies reveal significant differences in the average daily treatment costs for pressure injury patients (40). These disparities are primarily attributed to the severity of the injury, the type of treatment, and the level of the healthcare facility. Our study found that the use of silicone foam dressings for the prevention of sacral and heel pressure injury holds significant economic value (41). The direct treatment costs for the control group could be 4.2 times higher than those for the intervention group, with an average treatment cost per pressure injury case exceeding by 3.41 euros (42). It is important to note that these cost-effectiveness analyses are based solely on the hospital perspective and do not yet include societal costs such as patients’ work loss due to pressure injury, decreased quality of life, and additional consumption of community medical resources. There is a need for multicenter, large-sample health economic studies to further validate their economic value across different healthcare systems (34). Additionally, a comprehensive assessment model that includes societal costs should be established to provide more holistic evidence-based support for clinical decision-making and health policy formulation (43).

While this meta-analysis offers valuable insights to inform clinical decision-making, its findings warrant cautious interpretation due to several methodological limitations. First, although the international diversity of included studies enhances the potential generalizability of results, it introduces inherent variability in effect size estimates, stemming from differences in racial demographics, age distributions, and baseline physiological profiles across study populations. Such variability may obscure nuanced subgroup effects and complicate direct comparisons between cohorts. Second, methodological rigor was constrained by the absence of participant and assessor blinding across all included RCTs, coupled with limited reporting of allocation concealment (documented in only two studies). These omissions elevate the risk of performance bias (e.g., differential application of co-interventions) and detection bias—particularly relevant for visually assessed outcomes like pressure injury staging—thereby undermining the internal validity of individual study results and, by extension, the pooled analysis. Third, significant heterogeneity in baseline patient characteristics (e.g., comorbidity burden) and disease severity across included studies introduces substantial clinical variability, which may restrict the external validity of our conclusions. This heterogeneity limits the precision with which we can generalize findings to specific patient subgroups or clinical settings. To address these limitations and strengthen the evidence base, future research should prioritize large-scale, multicenter RCTs designed with standardized risk assessment protocols, rigorous control of key confounding variables (including nutritional status, mobility levels, and comorbidity management), and extended follow-up periods. Such studies would enable systematic evaluation of long-term intervention effects, enhance comparability across cohorts, and mitigate biases inherent in the current evidence, ultimately providing more robust guidance for clinical practice.

## Conclusion

In conclusion, this study advances the current evidence base through a focused, systematic analysis that confirms the efficacy of silicone foam dressings in reducing the incidence of pressure injuries specifically in the sacral and heel regions—anatomical sites of high clinical relevance that have been less comprehensively evaluated in prior meta-analyses, which often adopted broader scope across multiple body regions. Notably, while existing literature has intermittently highlighted the cost-effectiveness of silicone foam dressings, our analysis identifies a critical gap: such evaluations remain limited in scale and predominantly focus on short-term economic outcomes. This observation underscores a key distinction from earlier meta-analyses, which either omitted cost-related endpoints or lacked granularity in assessing their temporal dimensions. Given that pressure injury management entails sustained medical resource investment—with far-reaching implications for patient quality of life, functional outcomes, and societal burdens—our work emphasizes the need to expand the analytical framework beyond short-term metrics.

## Data Availability

The original contributions presented in the study are included in the article/supplementary material, further inquiries can be directed to the corresponding authors.
